# Anti-nucleocapsid antibodies enhance the production of IL-6 induced by SARS-CoV-2 N protein

**DOI:** 10.1038/s41598-022-12252-y

**Published:** 2022-05-16

**Authors:** Emi E. Nakayama, Ritsuko Kubota-Koketsu, Tadahiro Sasaki, Keita Suzuki, Kazuko Uno, Jun Shimizu, Toru Okamoto, Hisatake Matsumoto, Hiroshi Matsuura, Shoji Hashimoto, Toshio Tanaka, Hiromasa Harada, Masafumi Tomita, Mitsunori Kaneko, Kazuyuki Yoshizaki, Tatsuo Shioda

**Affiliations:** 1 Research Institute for Microbial Diseases and Center for Infectious Disease Education and Research, Osaka University, Suita, Osaka, 565-0871 Japan; 2 TANAKA Kikinzoku Kogyo K.K, Hiratsuka, Kanagawa 254-0076 Japan; 3 Division of Basic Research, Louis Pasteur Center for Medical Research, Kyoto, 606-8225 Japan; 4 MiCAN Technologies Inc., Kyoto, 615-8245 Japan; 5 Institute for Advanced Co-Creation Studies, Research Institute for Microbial Diseases, Osaka University, Suita, Osaka, 565-0781 Japan; 6 Trauma and Acute Critical Care Center, Osaka University, Suita, Osaka, 565-0871 Japan; 7 Osaka Prefectural Nakakawachi Emergency and Critical Care Center, Higashiosaka, Osaka, 678-0947 Japan; 8 Osaka Prefectural Hospital Organization Osaka Habikino Medical Center, Habikino, Osaka, 583-8588 Japan; 9 Yao Tokushukai General Hospital, Yao, Osaka, 581-0011 Japan; 10 Kobe Tokushukai Hospital, Kobe, Hyogo 655-0017 Japan; 11 Suita Tokushukai Hospital, Suita, Osaka, 565-0814 Japan; 12 Institute of Scientific and Industry Research, Osaka University, Ibaraki, Osaka, 567-0047 Japan

**Keywords:** Immunology, Microbiology, Diseases, Pathogenesis, Risk factors

## Abstract

A cytokine storm induces acute respiratory distress syndrome, the main cause of death in coronavirus disease 2019 (COVID-19) patients. However, the detailed mechanisms of cytokine induction due to severe acute respiratory syndrome coronavirus 2 (SARS-CoV-2) remain unclear. To examine the cytokine production in COVID-19, we mimicked the disease in SARS-CoV-2-infected alveoli by adding the lysate of SARS-CoV-2-infected cells to cultured macrophages or induced pluripotent stem cell-derived myeloid cells. The cells secreted interleukin (IL)-6 after the addition of SARS-CoV-2-infected cell lysate. Screening of 25 SARS-CoV-2 protein-expressing plasmids revealed that the N protein-coding plasmid alone induced IL-6 production. The addition of anti-N antibody further enhanced IL-6 production, but the F(ab’)^2^ fragment did not. Sera from COVID-19 patients also enhanced IL-6 production, and sera from patients with severer disease induced higher levels of IL-6. These results suggest that anti-N antibody promotes IL-6 production in SARS-CoV-2-infected alveoli, leading to the cytokine storm of COVID-19.

## Introduction

An outbreak of pneumonia in Wuhan, China, was first reported around December 2019. The new coronavirus, originally called 2019-nCoV, was then named severe acute respiratory syndrome coronavirus (SARS-CoV-2)^1^, and the disease was called coronavirus disease 2019 (COVID-19). The coronavirus is an enveloped virus with a circular lipid bilayer with a diameter of 100–160 nm. The surface of the particle is covered with spike (S) proteins, and the membrane contains membrane (M) and envelope (E) proteins. The internal genetic information is a single-stranded RNA with positive polarity, which is the longest viral RNA of approximately 30 kb long. The genomic RNA constitutes a nucleocapsid together with the N protein. The genomic RNA has a cap structure at the 5' end and poly A at the 3' end. At the 5' end of the genome is a leader sequence consisting of about 70b, downstream of which are RNA synthetase (ORF1a, 1b), S, E, M, and N genes, in that order, and other regions encoding small non-structural proteins^2^.

Common clinical manifestations of COVID-19 include fever, cough, and fatigue. Compared with moderate cases, severe cases more frequently have dyspnea, lymphopenia, and hypoalbuminemia, with higher levels of alanine aminotransferase, lactate dehydrogenase, C-reactive protein, ferritin, and D-dimer^3^.

Numerous cytokines and chemokines have been associated with the acute respiratory distress syndrome (ARDS) caused by SARS^4^ and SARS-CoV-2^5–7^. Among these cytokines, interleukin (IL)-6 is particularly important for predicting the disease severity^8–13^. Monocytes are one of the main sources of this cytokine at inflammatory sites^14^, and increased IL-6 expression in alveolar macrophages was reported in pneumonia patients, including those with severe COVID-19^15–17^. The virus can theoretically replicate in human macrophages and dendritic cells, triggering the aberrant production of proinflammatory cytokines/chemokines, if all components of viral binding and activation are available^18^. Regarding Middle East respiratory syndrome (MERS) coronavirus, another coronavirus that is less closely related to SARS-CoV-2 than SARS-CoV, it has been reported that the viral RNA levels increased in the culture supernatant for 3 days, but that the infectivity of the supernatant dropped during these 3 days^19^. Yilla et al. examined the replication of SARS-CoV in purified monocytes/macrophages, and found that SARS-CoV replicated poorly^20^, although preliminary electron microscopic studies demonstrated that SARS-CoV-like particles could enter the cells, possibly via phagocytosis. Despite the abortive infection of SARS-CoV-2^21,22^, which is characterized by infection without replication, SARS-CoV infection of human macrophages induced the expression of proinflammatory cytokines including IL-6^23^. These observations prompted us to investigate the mechanisms of the expression of IL-6 in monocyte-derived macrophages (MDM) infected with SARS-CoV-2. Here, we demonstrate the N protein-mediated induction of IL-6, which was further enhanced by specific antibodies against N protein.

## Results

### SARS-CoV-2-infected cell lysates induce IL-6 production from MDM

**Figure 1:**
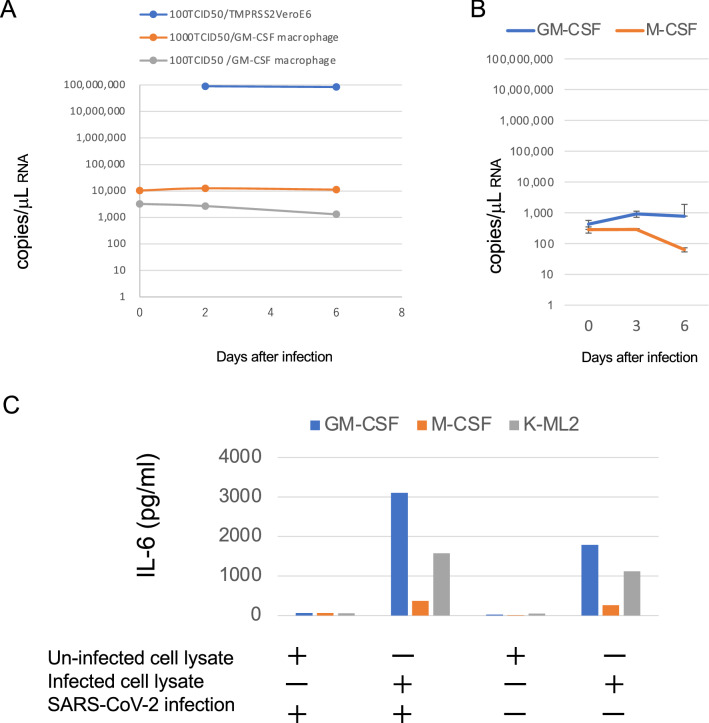
IL-6 production induced by SARS-CoV-2-infected cell lysate in monocyte-derived macrophages (MDM) and iPS-derived myeloid cells without productive infection. (**A**) Peripheral blood monocytes were differentiated into MDM by adding GM-CSF for 8 days. TMPRSS2/VeroE6 cells and MDM were infected with 1000 50% tissue culture infectious dose (TCID50) or 100 TCID50 of SARS-CoV-2 (JPN-TY-Wk-521 strain) and the viral RNA in the supernatants was measured by RT-PCR on the indicated days. (**B**) GM-CSF- or M-CSF- MDM were infected with 100 TCID50 of KNG-19-020 strain of SARS-CoV-2. (**C**) iPS-derived myeloid cells K-ML2 (gray) and GM-CSF- (blue) and M-CSF- (red) MDM were inoculated with the SARS-CoV-2 KNG-19-020 strain together with SARS-CoV-2-infected or uninfected cell lysate. Two days after infection, the IL-6 levels in the culture supernatants were measured by ELISA. The representative results of at least three independent experiments are shown.

We first examined whether or not peripheral blood MDM could be productively infected with SARS-CoV-2. Results showed virtually no SARS-CoV-2 growth in MDM differentiated with GM-CSF (Fig. 1A and S1A) or M-CSF (Fig. 1B). Production of IL-6 was also not observed after simple inoculation of SARS-CoV-2 (Fig. S2). These results were similar to those of a recent report^24–26^. We then examined the effects of SARS-CoV-2-infected cells on MDM as a model of SARS-CoV-2 infection in alveoli. Since MDM would contact components of epithelial cells in SARS-CoV-2-infected alveoli, we added freeze/thawed lysates of TMPRSS2/VeroE6 cells infected with SARS-CoV-2 to MDM together with SARS-CoV-2. Although the levels of SARS-CoV-2 RNA decreased over time (Fig. S2), elevated levels of IL-6 were detected in the supernatants of MDM incubated with SARS-CoV-2-infected cell lysate compared with those by adding uninfected cell lysate (Figs. 1C, S1B and S2). Three different strains of SARS-CoV-2 isolates were able to induce IL-6 (Fig. S2).

For further analysis, we tested K-ML2 cells as a substitute for MDM. K-ML2 cells are induced pluripotent stem (iPS) cell-derived myeloid cells expressing genetically engineered GM-CSF and M-CSF^27^, and they can be maintained and expanded easily as an established cell line. We can therefore use human myeloid cells without a blood donation from healthy donors and long incubation for differentiation into macrophages. Although K-ML2 cells lack the expression of ACE2 and TMPRSS2 required for entry of SARS-CoV-2, they were used for isolation, neutralization assay, and Fc-receptor-dependent antibody-dependent enhancement (ADE) assay of dengue virus^28^. As expected, the SARS-CoV-2-infected cell lysates could induce IL-6 production from K-ML2 cells, and further addition of SARS-CoV-2 was not required for IL-6 induction in both MDM and K-ML2 cells (Fig. 1C). These results suggested that a certain component in SARS-CoV-2-infected cells stimulated MDM and K-ML2 cells to produce IL-6.

### SARS-CoV-2 N protein induces IL-6 production more efficiently than S protein

**Figure 2:**
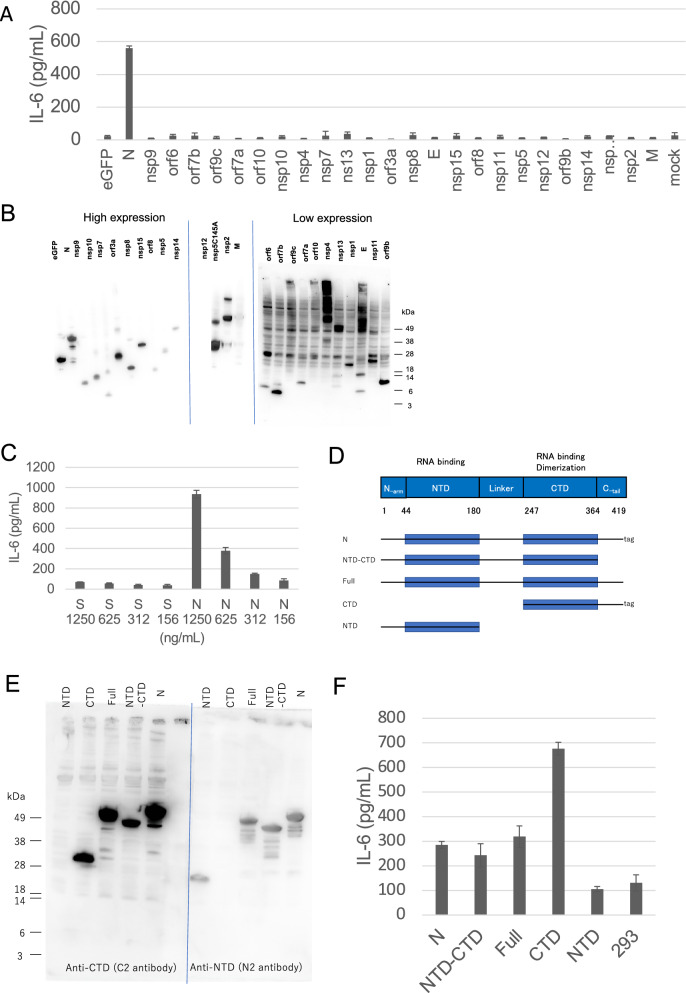
SARS-CoV-2 N protein, but not S protein, induced IL-6 production. The levels of IL-6 were measured by ELISA 2 days after treatment (**A**, **C**, and **F**). (**A**) K-ML2 cells were stimulated with the lysate of 293 T cells transfected with plasmids encoding each of the SARS-CoV-2 proteins. (**B**) The expression of each viral protein confirmed by western blot using the anti-Strep tag. The images from three membranes were combined. The original images with different exposure time are shown in Fig. S3. (**C**) K-ML2 cells were stimulated with serially diluted S or N protein produced using a baculovirus expression system. The mean and standard deviation of triplicate samples are shown. (**D**) A schematic diagram of full-length and truncated N proteins. (**E**) Anti-NTD (N2) antibody and anti-CTD (C2) antibody were used to visualize the full-length and truncated N proteins by western blot. The images from two membranes transferred from a single gel were combined. A blue vertical dividing line of two membranes is shown. The original images with different exposure time are shown in Fig. S3. (**F**) K-ML2 cells were stimulated with the cell lysates of 293 T cells expressing the full-length or truncated N proteins. Data are expressed as the mean and standard deviation of triplicate samples. The representative results of three independent experiments are shown.

We speculated that the SARS-CoV-2 S protein induced IL-6^11,29^. Addition of recombinant S protein at a final concentration of 1000 ng/mL did indeed induce IL-6 at a level comparable to that induced by the SARS-CoV-2-infected cell lysate (Fig. S1C). However, the level of S protein in the SARS-CoV-2-infected cell lysate used in our experiment was only 10 ng/mL, suggesting that a factor other than S protein was responsible for the IL-6 induction. Screening of 25 SARS-CoV-2 protein-expressing plasmids transfected into 293 T cells revealed that the N protein-coding plasmid alone induced IL-6 production (Figs. 2A, B and S3). Recombinant N protein also induced IL-6, and could induce much higher levels of IL-6 than the recombinant S protein (Fig. 2C).

To identify the specific domain in the N protein that is responsible for inducing IL-6 production, we constructed plasmids encoding truncated versions of the N protein (Figs. 2D, E and S3). The specific domain responsible for inducing IL-6 was located in the C-terminal domain (CTD) of the N protein, since IL-6-inducing activity was found in the cell lysate containing the CTD of the N protein (Fig. 2F). The N-terminal domain (NTD) of the N protein did not stimulate an increase in IL-6 from the basal level (Fig. 2F).

### N protein induces the production of multiple cytokines, including IL-8, from peripheral MDM

**Figure 3:**
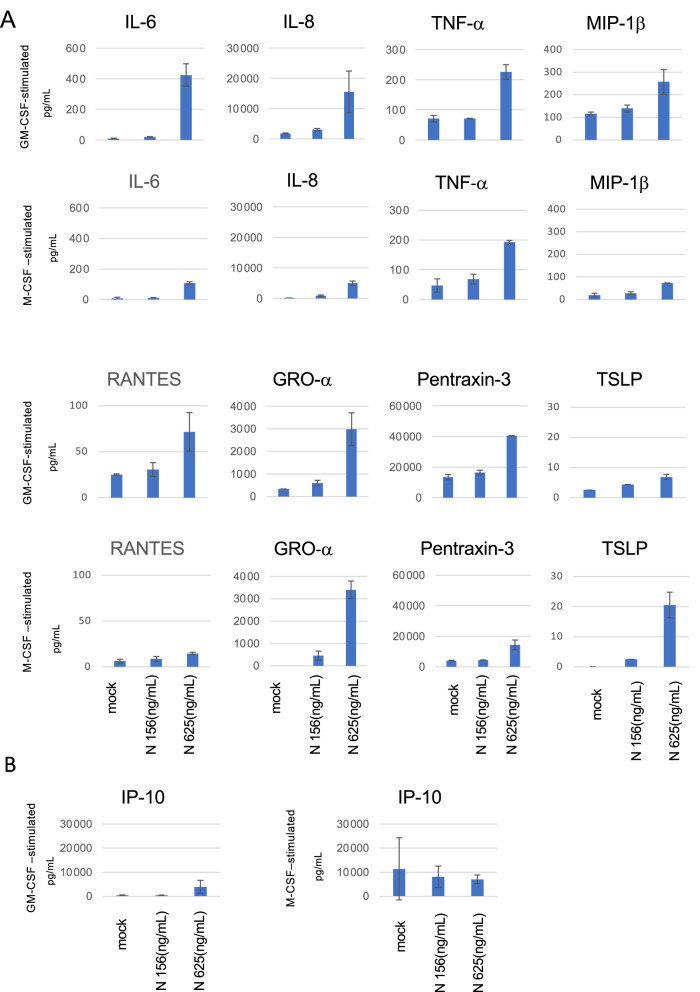
Profiles of cytokine production from macrophages (MDM) stimulated by N protein. MDM differentiated with GM-CSF or M-CSF were stimulated by 156 or 625 ng/mL of N proteins for 2 days. The cytokine levels were measured by a multiplex assay, as described in the Methods. Data are expressed as the mean and standard deviation of triplicate samples.

The levels of IL-6, IL-8, and TNF-α are known to be elevated in severe COVID-19 cases^30–32^. IL-8 is thought to be directly involved in pneumonia, since IL-8 attracts neutrophils. The culture supernatants of MDM 2 days after N protein stimulation were assayed for the levels of the cytokines and chemokines shown in Table S1. We found that the levels of multiple cytokines, including IL-6, IL-8, TNF-α, MIP-1β, RANTES, GRO α, pentraxin-3, and TSLP, were elevated after the addition of N protein in both GM-CSF-stimulated and M-CSF-stimulated MDM (Table S1). Higher levels of IL-6, IL-8, MIP-1β and RANTES were observed in cultures of GM-CSF-stimulated MDM than in M-CSF-stimulated MDM (Fig. 3A). In the case of IP-10, which was previously proposed as a predictive marker for severe disease in COVID-19^33^, an N protein-induced increase was observed only in GM-CSF-stimulated MDM, but not in M-CSF-stimulated MDM which expressed high basal levels (Fig. 3B). These findings indicated that MDM can be a source of cytokines in COVID-19 patients even without productive infection of SARS-CoV-2.

### Anti-N protein monoclonal antibodies enhance IL-6 production via the Fc-receptor

**Figure 4:**
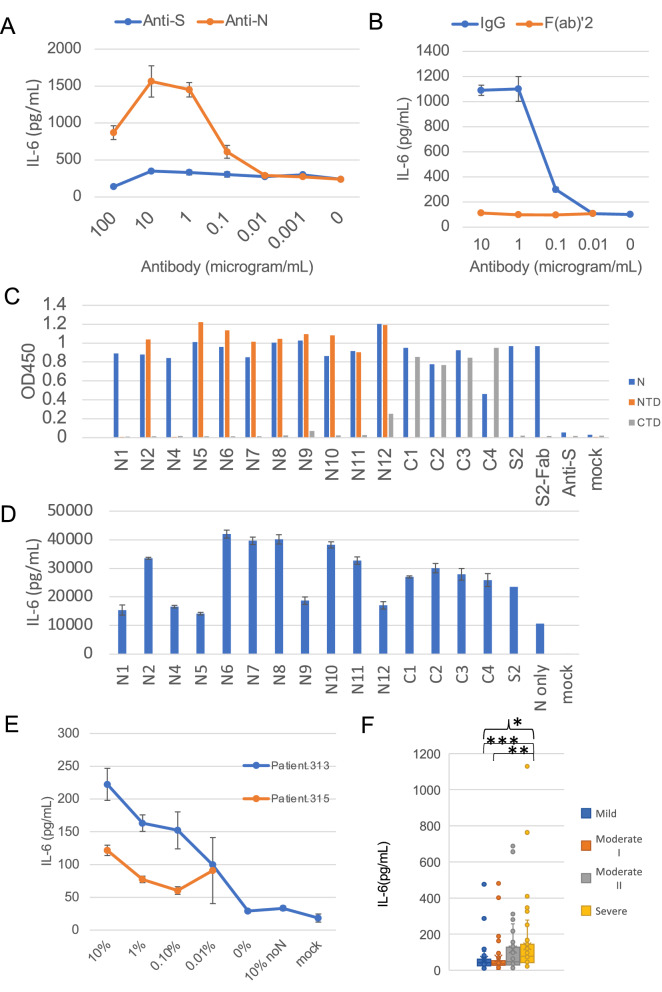
Anti-N antibodies and COVID-19 patient sera enhanced the IL-6 production induced by N protein. Levels of IL-6 were measured by ELISA 2 days after the addition of N protein, except for in panel C. (**A**) K-ML2 cells were stimulated with 156 ng/mL of N protein with escalating amounts of anti-S (blue) or anti-N (red) antibodies. (**B**) Anti-N antibody, S2, was digested by pepsin, and purified F(ab’)2 (red) or IgG (blue) was added to induce IL-6 production. Data are expressed as the mean and standard deviation of triplicate samples. (**C**) The levels of mouse mAbs bound to the full-length N protein (blue), NTD (Thr49-Gly175 fragment: red) or CTD (Lys248-Pro365 fragment: gray) were measured by an in-house ELISA. Anti-S mAb, clone 29-C7, was used as a negative control. (**D**) K-ML2 cells were stimulated with 3 μL of the cell lysate of N protein-expressing 293 T cells in the presence of 1 μg/mL of mAbs. Data are expressed as the mean and standard deviation of triplicate samples. The representative results of three independent experiments are shown. (**E**) K-ML2 cells were stimulated with 156 ng/mL of N protein with escalating amounts of serum from two patients, Patient 313 (blue) and Patient 315 (red). “0%” indicates the baseline production of IL-6 induced by N protein without patients’ serum. “10% noN” indicates the level of non-specific IL-6 induction from the addition of 10% patient serum from patient No. 313 without N protein. (**F**) The levels of IL-6 in the culture supernatants of K-ML2 cells cultured in the presence of 1% patient serum. Specimens were divided into four groups according to the disease severity at the time of blood collection. The criteria for COVID-19 severity in Japan were described in the Methods section. In brief, the criteria for mild, moderate I, moderate II, and severe are almost the same as those of mild, moderate, severe, and critical in the US NIH guideline. The center lines in the boxes and the boxes indicate the median and 25/75 percentiles, respectively. “*” denotes a statistically significant difference among the four groups (*p* < 0.0001) by the Kruskal–Wallis test. “**” and “***” denote a statistically significant difference between the moderate I and severe disease groups (*p* < 0.0001) and between the mild and severe disease groups (*p* < 0.0001), respectively, by the Mann–Whitney U test.

We added S2-2-5 monoclonal antibody (mAb) against N protein (S2) to neutralize the N protein that induced IL-6 production. Contrary to our expectation, the anti-N protein mAb enhanced IL-6 production (Fig. 4A). This enhancing activity was found to be Fc-receptor-dependent, since the F(ab’)2 form of the S2 mAb did not enhance IL-6 production (Fig. 4B). We then obtained 15 mAbs against N protein, as listed in Table S2, and tested their reactivity to the entire N protein, NTD, and CTD by enzyme-linked immunosorbent assay (ELISA). Four mAbs (C1–C4) reacted with the N protein and CTD, and seven mAbs (N2, N5, N6, N7, N8, N10, and N11) reacted with the N protein and NTD. Two mAbs (N1 and N4) reacted only with the N protein, and two mAbs (N9 and N12) reacted with the N protein and NTD, and weakly with the CTD (Fig. 4C). All four mAbs that bound to the CTD enhanced IL-6 production, while six of the nine mAbs that bound to the NTD enhanced IL-6 production (Fig. 4D). Interestingly, two mAbs (N1 and N4) lacking reactivity with both the CTD and NTD only weakly enhanced IL-6 production (Fig. 4D). Similar to the S2 mAb, the F(ab’)2 form of the S2 mAb retained reactivity to the N protein (Fig. 4C). These results suggested that the anti-N antibody enhanced the uptake of N protein into macrophages via the Fc-receptor.

### Patient sera from severe COVID-19 cases enhance the IL-6 production induced by N protein

To determine whether or not anti-N antibodies that are raised from actual SARS-CoV-2 infection can also enhance the IL-6 production induced by N protein, we added serially diluted patient sera to K-ML2 cells with N protein. We found that 0.01% to 10% of the COVID-19 patient sera clearly enhanced the IL-6 production induced by N protein (Fig. 4E). The addition of sera from five healthy donors did not affect the IL-6 production (Fig. S2). IL-8 levels were also enhanced by N protein in the presence of 1% of 24 COVID-19 patients’ sera (Fig. S4A). We then evaluated a total of 201 serum samples from 63 mild, 43 moderate I, 40 moderate II, and 55 severe COVID-19 cases. We found that sera from the severe cases induced significantly more IL-6 than those from the other cases (Fig. 4F). The levels of anti-N antibodies in the sera also tended to be elevated in the severe cases (Fig. S4B).

## Discussion

It is well-known that the course of COVID-19 varies among individuals, and that the medical condition of patients suddenly worsens 5–8 days after the onset of symptoms^15,34,35^. This disease course suggests the involvement of certain acquired immune responses to SARS-CoV-2, but the precise mechanisms of the rapid disease progression are not well understood. Several previous studies have shown that the anti-N antibody levels were positively correlated with disease severity^36,37^. In the present study, we showed that the N protein produced in infected epithelial cells, which were refractory to virus production, can induce IL-6 production from bystander macrophages or myeloid cells, and that antibodies against N protein enhanced this phenomenon.

Recently, Kawaciak et al. reported that both the S and N proteins of SARS-CoV-2 induced IL-6 in monocytes and macrophages by using 1000 ng/mL of recombinant proteins^38^. Zhang et al. previously showed that an 80-amino acid residue of the C terminus of the SARS N protein was required for IL-6 production via the transcription factor nuclear factor-kB^39^. During the preparation of our manuscript, Pan et al. reported that the CTD of the SARS-CoV-2 N protein interacts with NOD-, LRR- and pyrin domain-containing protein 3 (NLRP3) to activate inflammasomes and the NF-kB pathway^40^. Our results also showed the importance of the C terminal portion of the N protein for inducing IL-6. In addition, we clarified that the N protein has a higher potency to induce IL-6 than the S protein, and proposed that Fc-receptor-dependent and anti-N-antibody-dependent enhancement of IL-6 may be a trigger of cytokine storm.

ADE of coronavirus infection was first reported in feline infectious peritonitis virus^41^, and it has been thought to enhance the infection of SARS-CoV in macrophages via the Fc-receptor^42^. Phenomena similar to ADE have been observed in multiple SARS-CoV animal models. In a mouse model, attempts to create vaccines against SARS-CoV led to pulmonary immunopathology upon challenge with SARS-CoV^43,44^; these vaccines included inactivated whole viruses, inactivated viruses with adjuvant, a recombinant S protein, and a mouse hepatitis coronavirus-like particle vaccine, and the vaccinated mice showed increased eosinophilic proinflammatory pulmonary responses upon challenge. Maemura et al. suggested that the anti-S antibodies that elicit ADE of infection may not be involved in aberrant cytokine release by macrophages during SARS-CoV-2 infection^25^. In contrast, severe pneumonia was observed in mice vaccinated with N protein after challenge with SARS-CoV^45^. Based on these findings and our results, we propose that ADE of cytokine production from macrophages occurs even without productive infection.

Our proposed mechanism for the ADE of IL-6 production can explain why most of the previously developed vaccines against SARS-CoV and MERS viruses failed to elicit protective immunity against these viruses. Furthermore, our finding is consistent with the fact that the efficacy of inactivated vaccines against severe COVID-19 is lower than that of mRNA-based vaccines^46,47^. In addition, our model is also consistent with the fact that therapy with convalescent plasma, which contains anti-N antibodies, failed to improve the disease course in several studies^48–50^. Our results suggest that vaccination with an mRNA-based or adenovirus-based vaccine, which induces an anti-S protein immune response alone, would be highly recommended for those who have previously been infected with SARS-CoV-2, since the pre-existing anti-N protein antibody may be a risk factor for the development of a cytokine storm if they get infected with SARS-CoV-2 again. It would be safer to generate an inactivated whole virus vaccine in which N protein epitopes are engineered not to bind to enhancing anti-N antibodies.

It has been observed that pre-existing memory B cells to other human coronaviruses, such as OC43 and HKU-1^51^, might respond to SARS-CoV-2 rapidly^37,52,53^. Early high antibody responses are correlated with increased disease severity in both SARS^54^ and COVID-19^30,55–58^. As described above, the anti-N antibody levels are correlated with disease severity^36,37^. In addition, it is well-known that an age over 65 years is a risk factor for severe COVID-19^59^. Gorse et al. reported that the levels of antibodies to coronaviruses were higher in older adults than in younger adults^52^. Li et al. analyzed the anti-SARS-CoV-2-specific IgG levels in COVID-19 patients according to age, and found that the most significant difference in the levels of anti-N antibody during hospitalization was observed between those aged < 65 years and those aged > 65 years^53^. Our results may explain why elderly people, who are more likely to have been previously exposed to human common cold coronaviruses, experience more severe COVID-19 than the younger generation.

Regarding the treatment of patients, steroids, anti-IL-6 receptor antibody^12,60^, and anti-JAK treatment^61^ are recommended to suppress excessive immune responses. NLRP3 signal inhibitors were also proposed as candidate drugs^40^. However, these treatments increase the risk of other infections, including those by bacteria and fungi, since they suppress the host immune defense. Furthermore, it has been reported that complement-mediated thrombotic microvascular injury in the lung may contribute to atypical ARDS features of COVID-19^62^. Complement activation was found in multiple organs of severe COVID-19 patients in several studies^63,64^. Kang et al. suggested that a monoclonal antibody against N protein can compromise the N protein-induced complement hyperactivation^65^. In the present study, we used 16 mAbs against N protein, and found that five antibodies did not enhance IL-6 production while 11 did. These five antibodies (N1, N4, N5 N9, and N12) may be useful as starting materials for novel treatment strategies for COVID-19. Although further studies are necessary to establish treatments for COVID-19 by suppressing IL-6, macrophage stimulation by N protein should be avoided to prevent the development of cytokine storm.

The present study has several limitations. We used peripheral blood monocyte-derived macrophages that were differentiated with either GM-CSF or M-CSF and iPS-derived myeloid cells as models of alveolar macrophages. Autologous human sera were previously used for differentiation of peripheral blood monocyte into macrophages^66^. It is thus necessary to identify which differentiation strategy best imitates the actual environment in alveoli. Experiments using alveolar macrophages recovered from broncho-alveolar lavage are also needed to strengthen the conclusion. The cytokine profiles from tissue-resident alveolar macrophages induced by N protein should be investigated in future studies.

## Methods

### Human materials

This study followed the principles of the Declaration of Helsinki, and was approved by the institutional review board of Osaka University Hospital (No. 885). Informed consent for the collection of blood samples was obtained from the patients or their relatives. The use of left-over specimens of daily tests after anonymization was approved by the institutional review board of Habikino Medical Center (150-7), Louis Pasteur Center for Medical Research (LPC29), and Tokushukai Hospital (TGE01547). A brief summary of the protocol was disclosed. The use of human blood from healthy volunteers in this study was approved by the Research Ethics Committee of the Research Institute for Microbial Diseases, Osaka University, Japan (No. 2021-3).

### Animals

The animal study was carried out in accordance with the recommendations in the guide for the care and use of laboratory animals of Osaka University, Japan. Our protocol was approved by the Committee on the Ethics of Animal Experiments of the Research Institute for Microbial Diseases, Osaka University, Japan (No. R01-17-0). All procedures in this animal experiment were conducted in a manner to avoid or minimize discomfort, distress, or pain to the animals according to the ARRIVE guidelines (https://arriveguidelines.org/) and the approved operating procedures and guidelines of the Research Institute for Microbial Diseases of Osaka University.

### Viruses and cells

SARS-CoV-2 (KNG19-020) was kindly supplied by Dr. Tomohiko Takasaki of the Kanagawa Prefectural Institute of Public Health. SARS-CoV-2 (JPN-TY-WK-521) was obtained from the National Institute for Infectious Diseases, Japan. Clinical isolates hCoV-19/Japan/OIPH14/2020 and hCoV-19/Japan/OIPH21/2020^67^ were propagated in TMPRSS2/VeroE6 cells^68^ that were obtained from the National Institutes of Biomedical Innovation, Health and Nutrition, Japanese Collection of Research Bioresources Cell Bank, Japan. K-ML2 cells were established as described previously^28^ and maintained in minimum essential medium supplemented with 10% fetal calf serum. Peripheral blood mononuclear cells were obtained from the blood buffy coats of healthy donors by Ficoll-Paque density gradient centrifugation, then plated in 24-well MULTIWELL PRIMARIA plates (Becton Dickinson, Franklin Lakes, NJ) containing RPMI 1640 supplemented with 10% fetal calf serum. Monocytes were differentiated into macrophages for 8 days in the presence of 100 ng/mL of GM-CSF (PeproTECH, Rocky Hill, NJ) or 50 ng/mL of M-CSF (PeproTECH). To prepare SARS-CoV-2 infected cell lysates, TMPRSS2/VeroE6 cells were infected with SARS-CoV-2 at a multiplicity of infection of 0.01, and the cells were harvested 16 h after infection. Subsequently, 1.6 × 10^5^ infected cells were suspended in 200 µL of phosphate-buffered saline (PBS) and frozen at − 20 °C. After thawing, 10 µL of suspended cell lysate was added to 100 µL of culture medium in each well containing macrophage with or without SARS-CoV-2 and diluted serum. After 4 h of incubation, macrophages were washed once with culture medium, then 500 µL of fresh medium was added, and the cells were cultivated for 6 days at 37 °C under an atmosphere of 5% CO_2_.

### Real-time RT-PCR

Viral RNA was extracted from 140 µL of culture supernatant using the QIAamp Viral RNA Mini Kit (QIAGEN, Hilden, Germany) according to the manufacturer’s instructions. Real-time quantitative reverse transcription PCR (RT-qPCR) assays were performed using the One Step TB Green PrimeScript RT-PCR kit II (Takara, Shiga, Japan) and the SYBR Green assay 2 primer sets designed by MilliporeSigma (https://www.sigmaaldrich.com/US/en/technical-documents/protocol/research-and-disease-areas/immunology-research/ncov-coronavirus). The components of the RT-qPCR reaction mixture were as follows: 6.25 µL of 2 × One Step SYBR RT-PCR Buffer 4, 0.25 µL of forward primer (10 µM), 0.25 µL of reverse primer (10 µM), 0.25 µL of ROX reference Dye II (50 ×), 2.5 µL of deionized water, 0.5 µL of PrimeScript 1 step Enzyme Mix 2, and 2.5 µL of template RNA. Reverse transcription was performed at 42 °C for 5 min, followed by denaturation at 95 °C for 10 min, and 40 amplification cycles at 95 °C for 5 s and 60 °C for 34 s. A Quant Studio 3 Real-Time PCR System (Life Technologies, Carlsbad, CA) was used for the analysis.

### IL-6 measurement

Serially diluted S protein (Spike S1 + S2 ECD-His Recombinant Protein, 40589-V08B1, Sino Biological, Beijing, China), N protein (40588-V08B or 40588-V07E, Sino Biological), or 10 µL of cell lysate suspended in 100 µL of culture medium was added to 100 µL of the K-ML2 cell suspension or monocyte-derived macrophages with 100 µL of culture medium and incubated at 37 °C for 4 h. Then, 500 µL of fresh medium was added to the wells. Two days later, the culture supernatants were harvested, and the levels of IL-6 were measured by an enzyme-linked immunoassay (ELISA MAX Deluxe Set Human IL-6, BioLegend, San Diego, CA).

### Plasmid construction and transfection

We used standard molecular biology techniques for cloning and plasmid construction. Most of the plasmid constructs were generated in the pLVX-EF1alpha-SARS-CoV-2-N-2xStrep-IRES-Puro vector backbone (for more details, see Table S3). A standard transfection reagent, TransIT-293 transfection reagent (V2704, Takara), was used.

### Western blot

Plasmid-transfected 293 T cells (6 × 10^5^ cells) were lysed in 100 μL of lysis buffer (50 mM Tris–HCl at pH 7.5, 150 mM NaCl, 1% Nonidet P-40, and 0.5% sodium deoxycholate). Proteins in the lysates were subjected to sodium dodecyl sulfate–polyacrylamide gel electrophoresis. The proteins in the gel were then electrically transferred to a membrane (Immobilion; Millipore, Billerica, MA). Blots were blocked and probed with N2 mAb (HM1057, EastCoast Bio, Maryland Heights, MO) or C2 mAb (CV15, CerTest Biotec, Zaragoza, Spain) overnight at 4 °C. The blots were then incubated with peroxidase-linked anti-mouse IgG (H + L), and the bound antibodies were visualized with a Chemi-Lumi One chemiluminescent kit (Nacalai Tesque, Kyoto, Japan).

### Immunization, fusion, and selection of mAb

SARS-CoV-2 (KNG19-020) was propagated in TMPRSS2/VeroE6 cells (JCBR1819) and purified by sucrose gradient centrifugation^69^. Concentrated virus was then exposed to ultraviolet light (0.6 J/cm^2^) to inactivate the virus. We confirmed that the virus had completely lost its infectivity by this method. BALB/c mice (4 weeks old, female) were intraperitoneally immunized three times by the inactivated virion (corresponding to 3.8 × 10^7^ TCID50/mouse) with adjuvant (1st immunization: Freund’s complete adjuvant (WAKO, Tokyo, Japan); 2nd and 3rd immunization: Freund’s incomplete adjuvant (WAKO)). Three days after the last immunization, splenic cells from the mice were used to prepare hybridomas. The hybridoma-producing mAbs were generated as described previously^70^ using mouse myeloma PAI cells. The antibodies secreted by the hybridomas were screened by an indirect immunofluorescence assay using SARS-CoV-2-infected cells. SARS-CoV-2-infected and mock-infected TMPRSS2/VeroE6 cells were cultured for 18 h, fixed with 7% formaldehyde-PBS for 30 min, and then permeabilized with 1% Triton X-100 PBS for 5 min. The cells were incubated with hybridoma culture supernatant at 37 °C for 1 h, followed by incubation with goat anti-mouse IgG conjugated to Alexa Flour 488 (1:1000; Invitrogen, Carlsbad, CA) for 30 min at 37 °C. The cells were observed under a fluorescence microscope (ECLIPSE Ti2, Nikon, Tokyo, Japan). The isotype of the antibodies was determined by an IsoStrip mouse Monoclonal Antibody Isotyping kit (Roche, Mannheim, Germany). The target of the antibodies was determined by the staining patterns using an indirect immunofluorescence assay and the reactivity to N protein-expressing cells.

### ELISA for S protein

The amount of S protein in the SARS-CoV-2-infected cell lysates was measured by a SARS-CoV-2 Spike Protein ELISA kit (E-EL-E605, Elabscience, Houston, TX) according to the manufacturer’s instructions.

### In-house ELISA of anti-N antibodies

Ninety-six-well flat-bottom microplates were coated with 100 ng/well of N protein (40588-V08B, Sino Biological), NTD (40588-V07E10, Sino Biological), or CTD (40588-V07E5, Sino Biological) in 50 μL of carbonate-bicarbonate buffer (C-3041, Sigma, St. Louis, MO), and incubated at 4 °C overnight. After washing with 0.05% Tween 20 in PBS (PBS-T), wells were blocked with 200 μL of a 25% solution of BlockAce for 1 h at room temperature. After washing with PBS-T, 100-times diluted patient serum with PBS-T or mouse mAb (1 μg/mL), as listed in Table S2, was added and incubated for 1 h at room temperature. After washing with PBS-T, 50 μL of the secondary antibody solution of peroxidase-conjugated AffiniPure alpaca anti-human IgG (H + L) (609-035-213, Jackson ImmunoResearch, Pennsylvania, PA) or peroxidase-labeled goat anti-mouse IgG (H + L) (5220–0341, CeraCare, Milford, MA) was added and incubated for 1 h at room temperature. A TMB substrate kit (34021, Thermo Fisher Scientific, Waltham, MA) was used for colorimetric detection, and the optical density at 450 nm was measured by a Multigrading Microplate Reader (SH-9500Lab, Corona, Hitachinaka, Ibaraki, Japan).

### Multiplex cytokine measurement

Cytokine and chemokine biomarkers were quantified using the Bio-Plex 200 multiplex cytokine array system (Bio-Rad Laboratories, Hercules, CA) according to the manufacturer’s instructions. We simultaneously quantified cytokines, chemokines, and soluble receptors using the Bio-Plex Human Cytokine 27-plex Panel (IL-1β, IL-1Rα, IL-2, IL-4, IL-5, IL-6, IL-7, IL-8, IL-9, IL-10, IL-12 (p70), IL-13, IL-15, IL-17A, eotaxin, basic fibroblast growth factor, G-CSF, GM-CSF, IFN-γ, IP-10, MCP-1, MIP-1α, platelet-derived growth factor-BB, MIP-1α, RANTES, TNF-α, and vascular endothelial growth factor; Bio-Rad Laboratories), the Inflammation Panel (a proliferation-inducing ligand (APRIL), B-cell activation factor, soluble CD30, soluble CD163, chitinase, soluble glycoprotein 130, IFN-α2, IFN-β, IFN-γ, IL-2, IL-8, soluble IL-6 receptor α, IL-10, IL-11, IL-12 (p40), IL-12 (p70), IL-19, IL-20, IL-22, IL-26, IL-27, IL-28A, IL-29, IL-32, IL-34, IL-35, lymphotoxin-like inducible protein that competes with glycoprotein D for herpesvirus entry on T cells (LIGHT), matrix metalloproteinase (MMP)-1, MMP-2, MMP-3, osteocalcin, osteopontin, pentraxin-3, soluble TNF receptor 1, soluble TNF receptor 2, thymic stromal lymphopoietin, and TNF-like weak inducer of apoptosis; Bio-Rad Laboratories), and the Bio-Plex pro (hepatocyte growth factor, IL-18, TNF-related apoptosis-inducing ligand, IL-2 receptor α, M-CSF, growth-related oncogene α, MCP-3, and monokine induced by IFN-γ Bio-Rad Laboratories. Data acquisition and analysis were performed using Bio-Plex Manager software version 5.0 (Bio-Rad Laboratories).

### Patient sera

The disease severity of patients was determined at hospital admission according to The Guideline for Medical Treatment of COVID-19 in Japan (https://www.mhlw.go.jp/content/000785119.pdf).

Briefly, patients with “mild” illness showed one or some of the signs and symptoms of COVID-19 (e.g., fever, cough, sore throat, malaise, headache, muscle pain, nausea, vomiting, diarrhea, and loss of taste and smell), but lacked shortness of breath, dyspnea on exertion, and abnormal imaging findings. “Moderate I” cases showed evidence of lower respiratory disease with a percutaneous oxygen saturation (SpO2) of > 93% on room air, and were compatible with “moderate illness” described in the Coronavirus Disease 2019 (COVID-19) Treatment Guidelines of the National Institutes of Health (NIH; https://www.covid19treatmentguidelines.nih.gov). “Moderate II” cases were supported with non-invasive mechanical ventilation or supplemental oxygen (including high-flow oxygen devices), and were compatible with “severe illness” described in the NIH guidelines. “Severe” cases were admitted into the intensive care unit or supported with invasive mechanical ventilation or extracorporeal membrane oxygenation, and were compatible with “critical illness” described in the NIH guidelines. In Japan, invasive ventilation was not applicable for several terminal cases. Aliquots of patient sera were collected from the leftover specimens of daily tests on Osaka University and Osaka Prefectural Nakakawachi Emergency and Clinical Care Center (March to August, 2020), Osaka Habikino Medical Center (April, 2020), Kobe Tokushukai Hospital (November to December, 2020), Suita Tokushukai Hospital (January to February, 2021), and Yao Tokushukai General Hospital (August 2020 to February 2021) at the time of admission to the hospitals and kept at − 80 °C until use. The median age among the 63 mild, 43 moderate I, 40 moderate II, and 55 severe cases was 57 (interquartile range, 37–72.5), 70 (56.5–80.75), 75.5 (58.25–85), and 69 (58.25–79) years, respectively. The number of males was 33 (52.4%), 22 (51.2%), 23 (57.5%), and 41 (74.5%), respectively.

### Quantification and statistical analysis

The numbers of repetitions of specific experiments are shown in the figure legends. For multiple comparisons, statistical analysis was performed by the Kruskal–Wallis and Mann Whitney U tests (GraphPad Prism version 9.0.2, GraphPad Software, San Diego, CA) where applicable (Fig. 4F).

## Supplementary Information

Supplementary Information.

## Data Availability

All data generated during this study are included in this published article and its supplementary information files.
