# Supplementary material for: Anti-nucleocapsid antibodies enhance the production of IL-6 induced by SARS-CoV-2 N protein
Source: Sci Rep. 2022 May 16;12:8108. doi: 10.1038/s41598-022-12252-y (PMC9109953; doi:10.1038/s41598-022-12252-y)
Supplement: Supplementary file 1 — Supplementary Information. [file 41598_2022_12252_MOESM1_ESM.pdf]

Table S1. Cytokine stimulation in N protein treated monocyte-derived macrophages.

	Stimulation	
Cytokines	GM-CSF	M-CSF
**IL-1b**	→	↑
**IL-1rα**	→	→
**IL-2**	↑	→
**IL-4**	↑	→
**IL-5**	↑	ND
**IL-6**	↑↑	↑
**IL-7**	→	→
**IL-8**	↑	↑↑
**IL-9**	→	↑
**IL-10**	→	→
**IL-12(p70)**	ND	ND
**IL-13**	→	→
**IL-15**	ND	ND
**IL-17A**	↑	→
**Eotaxin**	→	→
**FGF basic**	→	→
**G-CSF**	→	→
**GM-CSF**	added	→
**IFN-γ**	→	→
**IP-10**	↑	→
**MCP-1(MCAF)**	→	→
**MIP-1α**	↑	→
**PDGF-bb**	→	→
**MIP-1β**	↑	↑
**RANTES**	↑	↑
**TNF-α**	↑	↑
**VEGF**	ND	ND
**HGF**	→	(↓)
**IL-18**	ND	ND
**TRAIL**	↑	→
**IL-2Rα**	ND	ND
**M-CSF**	→	added
**GRO-α**	↑	↑↑
**MCP-3**	→	→
**MIG**	↑	→
**APRIL/TNFSF13**	→	→
**BAFF/TNFSF13B**	↑	→
**sCD30/TNFRSF8**	ND	ND
**sCD163**	→	→
**Chitinase 3-like 1**	→	→
**gp130/sIL-6Rb**	ND	ND
**IFN-α2**	ND	ND
**IFN-β**	ND	ND
**IL-6Rα**	→	→
**IL-10**	ND	↑
**IL-11**	→	→
**IL-12(p40)**	ND	ND
**IL-19**	ND	ND
**IL-20**	→	→
**IL-22**	↑	ND
**IL-26**	→	→
**IL-27(p28)**	ND	ND
**IL-28A/IFN-λ2**	→	ND
**IL-29/IFN-l1**	↑	ND
**IL-32**	ND	ND
**IL-34**	→	→
**IL-35**	ND	ND
**LIGHT/TNFSF14**	↑	ND
**MMP-1**	↑	ND
**MMP-2**	ND	ND
**MMP-3**	ND	ND
**Osteocalcin**	ND	ND
**Osteopontin(OPN)**	→	→
**Pentraxin-3**	↑	↑
**sTNF-R1**	→	→
**sTNF-R2**	→	→
**TSLP**	↑	↑↑
**TWEAK/TNFSF12**	→	→

↑↑: >2-fold increase at 156 and 625 ng/mL

↑: >2-fold increase at 625ng/mL

→: <2-fold increase

ND: not detected

GM-CSF: granulocyte macrophage colony stimulating factor

M-CSF:macophage colony stimulating factor

IL:interleukin

IFN: interferon

FGF:fibloblast growth factor

IP: interferon inducible protein

MCP: monocyte chemoatractant protein

MIP:macrophage imflammatory protein

PDGF:platelet-derived growth factor

RANTES:regulated on activation, normal T cell expressed and secreted

TNF: tumor necrosis factor

VEGF:vascular endothelial growth factor

HGF:hepatocyte growth factor

TRAIL:TNF-related apoptosis inducing ligand

GRO:growth-regulated oncogene product

MIG:monokine induced by INF γ

APRIL:A proliferation-inducing ligand

TNFSF:tumor necrosis factor ligand superfamily

BAFF:B-cell activating factor

LIGHT:lymphotoxin-related inducible ligand that competes for glycoprotein D binding to herpesvirus entry mediator on T cells

MMP:matrix metalloproteinase

TSLP:thymic stromal lymphopoietin

TWEAK:tumor necrosis factor-like weak inducer of apoptosis

Table S2. List of monoclonal antibodies

	Clone	Subclass	Manufacturer
N1	HM1054	IgG2b	EastCoastBio
N2	HM1057	IgG1	EastCoastBio
N4	3851	IgG2b	Virostat
N5	A72061	IgG1	BiosPacific
N6	A03092	IgG2a	BiosPacific
N7	A03090	IgG2a	BiosPacific
N8	A03091	IgG2a	BiosPacific
N9	HM1058	IgG1	EastCoastBio
N10	HM1063	IgG2a	EastCoastBio
N11	HM1065	IgG2a	EastCoastBio
N12	A72063	IgG1	BiosPacific
S2	S2-2-5-D6-3	IgG1	RIMD
C1	CV10	IgG2a	Certest
C2	CV15	IgG1	Certest
C3	B3449M	IgG1	MeridianLifescience
C4	B3451M	IgG1	MeridianLifescience

Table S3. List of plasmids

	Catalog No.	Name
1	141395	pLVX-EF1alpha-eGFP-2xStrep-IRES-Puro
2	141391	pLVX-EF1alpha-SARS-CoV-2-N-2xStrep-IRES-Puro
3	141375	pLVX-EF1alpha-SARS-CoV-2-nsp9-2xStrep-IRES-Puro
4	141387	pLVX-EF1alpha-SARS-CoV-2-orf6-2xStrep-IRES-Puro
5	141389	pLXV-EF1alpha-2xStrep-SARS-CoV-2-orf7b-IRES-Puro
6	141393	pLXV-EF1alpha-2xStrep-SARS-CoV-2-orf9c-IRES-Puro
7	141388	pLVX-EF1alpha-SARS-CoV-2-orf7a-2xStrep-IRES-Puro
8	141394	pLVX-EF1alpha-SARS-CoV-2-orf10-2xStrep-IRES-Puro
9	141376	pLVX-EF1alpha-SARS-CoV-2-nsp10-2xStrep-IRES-Puro
10	141369	pLVX-EF1alpha-SARS-CoV-2-nsp4-2xStrep-IRES-Puro
11	141373	pLVX-EF1alpha-SARS-CoV-2-nsp7-2xStrep-IRES-Puro
12	141379	pLVX-EF1alpha-SARS-CoV-2-nsp13-2xStrep-IRES-Puro
13	141367	pLVX-EF1alpha-SARS-CoV-2-nsp1-2xStrep-IRES-Puro
14	141383	pLVX-EF1alpha-SARS-CoV-2-orf3a-2xStrep-IRES-Puro
15	141374	pLVX-EF1alpha-SARS-CoV-2-nsp8-2xStrep-IRES-Puro
16	141385	pLVX-EF1alpha-SARS-CoV-2-E-2xStrep-IRES-Puro
17	141381	pLVX-EF1alpha-SARS-CoV-2-nsp15-2xStrep-IRES-Puro
18	141390	pLVX-EF1alpha-SARS-CoV-2-orf8-2xStrep-IRES-Puro
19	141377	pLVX-EF1alpha-SARS-CoV-2-nsp11-2xStrep-IRES-Puro
20	141370	pLVX-EF1alpha-SARS-CoV-2-nsp5-2xStrep-IRES-Puro
21	141378	pLVX-EF1alpha-SARS-CoV-2-nsp12-2xStrep-IRES-Puro
22	141392	pLVX-EF1alpha-SARS-CoV-2-orf9b-2xStrep-IRES-Puro
23	141380	pLXV-EF1alpha-2xStrep-SARS-CoV-2-nsp14-IRES-Puro
24	141371	pLVX-EF1alpha-SARS-CoV-2-nsp5-C145A-2xStrep-IRES-Puro
25	141368	pLVX-EF1alpha-SARS-CoV-2-nsp2-2xStrep-IRES-Puro
26	141386	pLVX-EF1alpha-SARS-CoV-2-M-2xStrep-IRES-Puro

S1

A

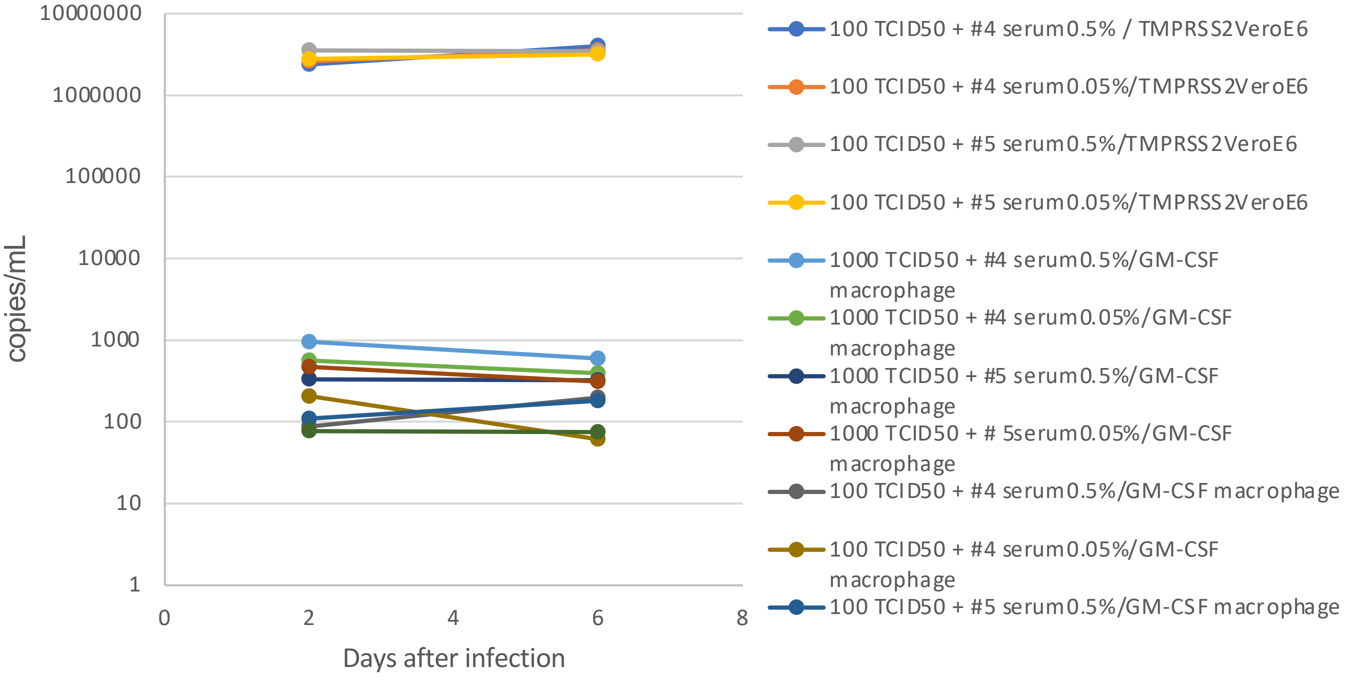

10000000
1000000
100000
10000
1000
100
10
1
copies/mL
0
2
4
6
8
Days after infection
100 TCID50 + #4 serum0.5% / TMPRSS2VeroE6
100 TCID50 + #4 serum0.05%/TMPRSS2VeroE6
100 TCID50 + #5 serum0.5%/TMPRSS2VeroE6
100 TCID50 + #5 serum0.05%/TMPRSS2VeroE6
1000 TCID50 + #4 serum0.5%/GM-CSF macrophage
1000 TCID50 + #4 serum0.05%/GM-CSF macrophage
1000 TCID50 + #5 serum0.5%/GM-CSF macrophage
1000 TCID50 + # 5serum0.05%/GM-CSF macrophage
100 TCID50 + #4 serum0.5%/GM-CSF macrophage
100 TCID50 + #4 serum0.05%/GM-CSF macrophage
100 TCID50 + #5 serum0.5%/GM-CSF macrophage

B

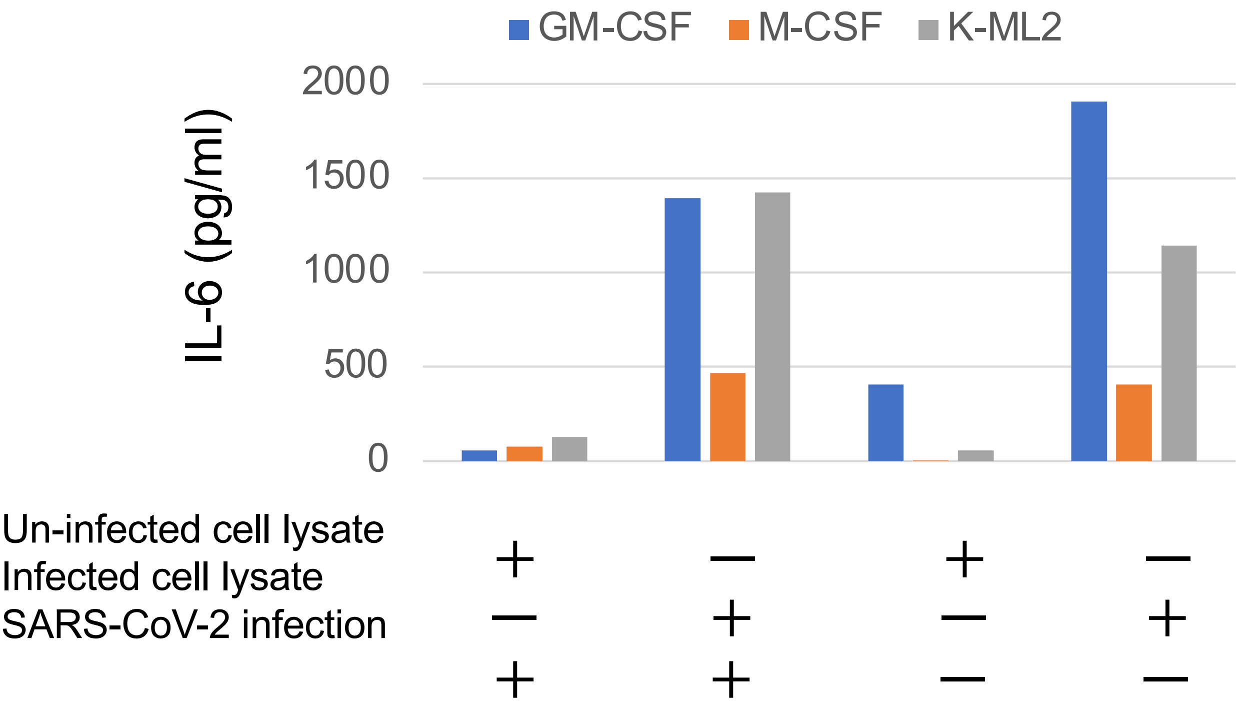

GM-CSF
M-CSF
K-ML2
2000
1500
1000
500
0
IL-6 (pg/ml)
Un-infected cell lysate
Infected cell lysate
SARS-CoV-2 infection
+ — +
— + +
+ — —
— + —

C

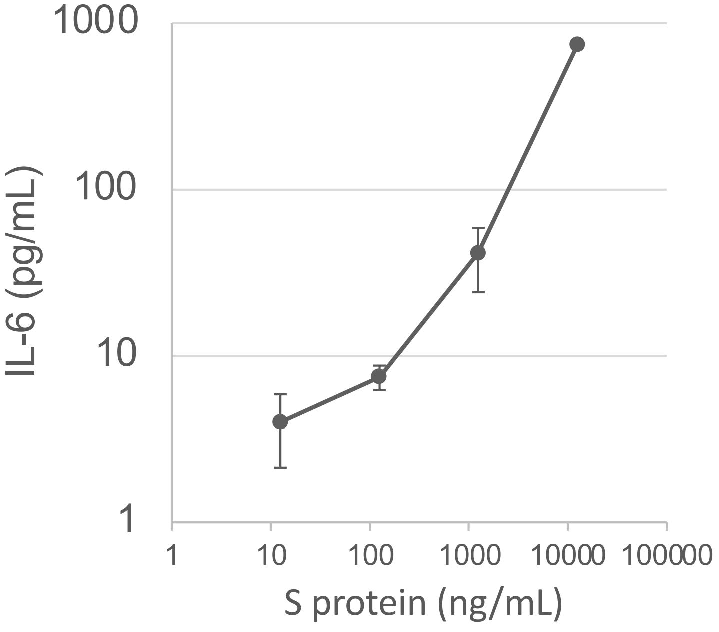

1000
100
10
1
IL-6 (pg/mL)
1
10
100
1000
10000
100000
S protein (ng/mL)

S2

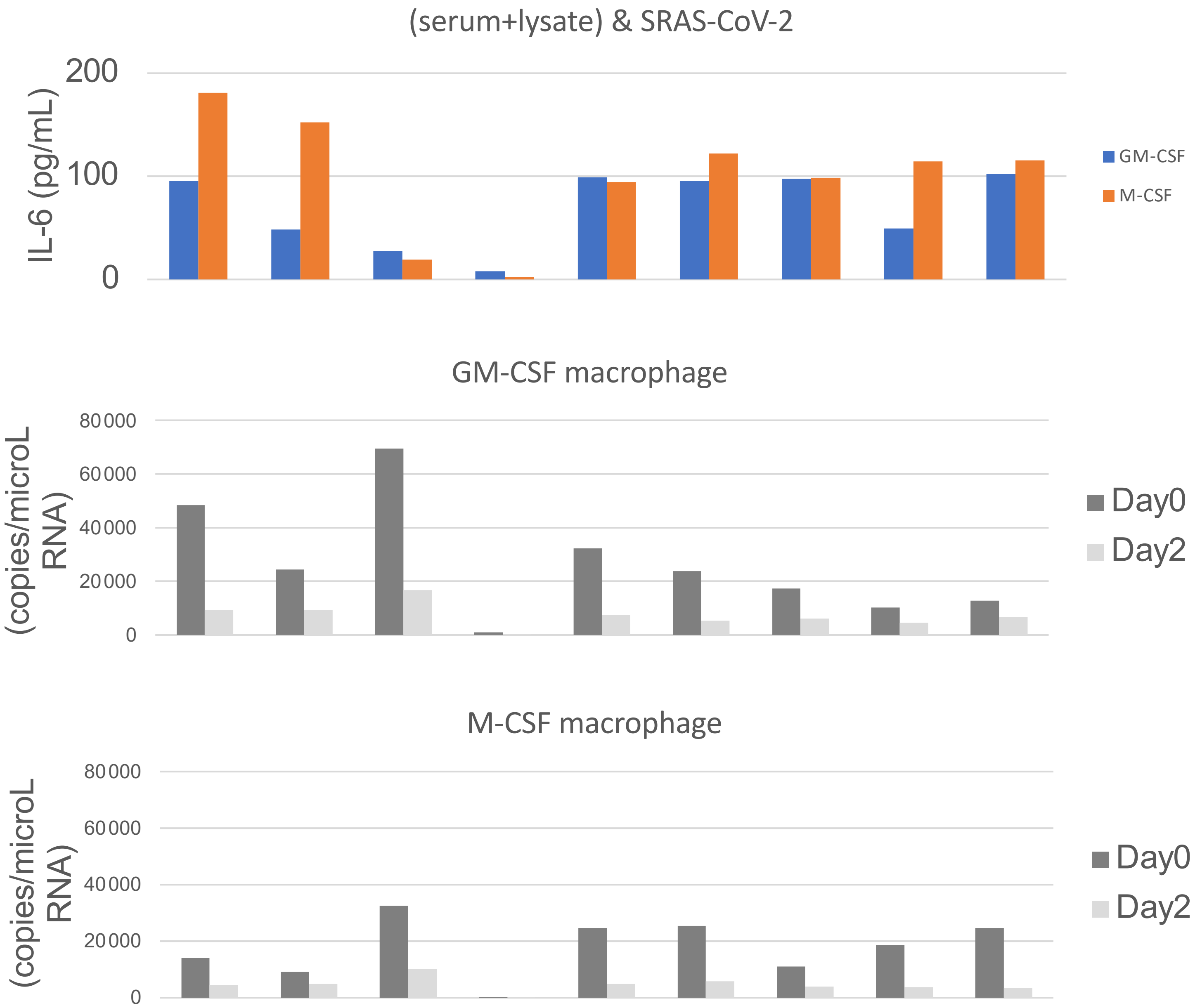

Serum	(-)	(-)	(-)	(-)	HD 2	HD 1	HD 3	HD 4	HD 5
lysate	(+)	A.1	B.1.1.248	(-)	(+)	(+)	(+)	(+)	(+)
over-infection	(+)	(+)	(+)	(+)	(+)	(+)	(+)	(+)	(+)

(+) KNG19-020 strain HD: Healthy donor

S3A

30sec

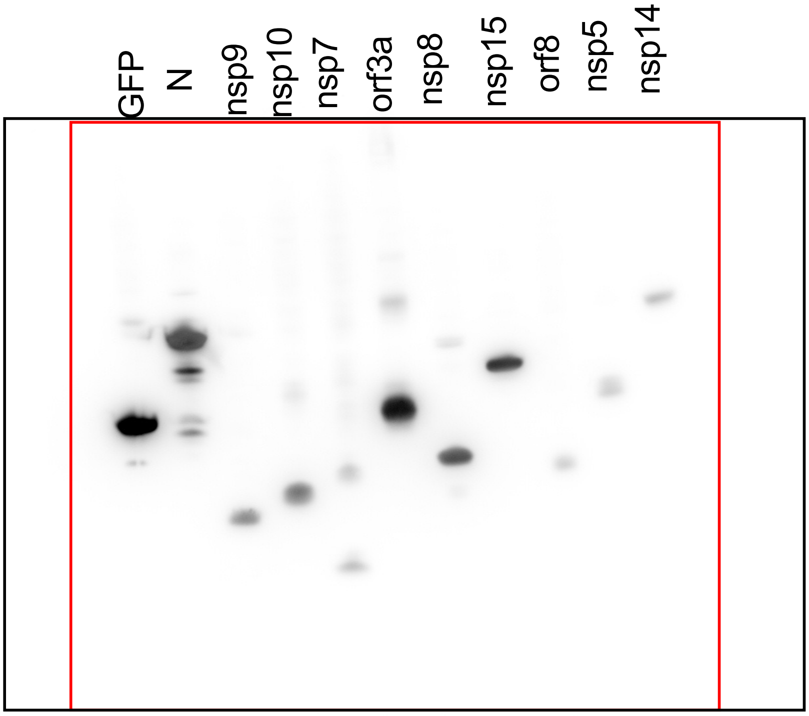

3min

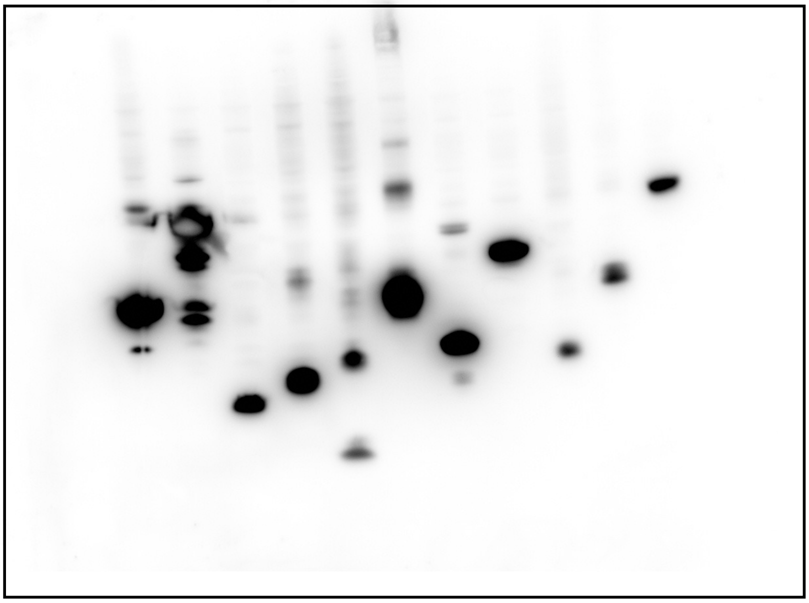

10min

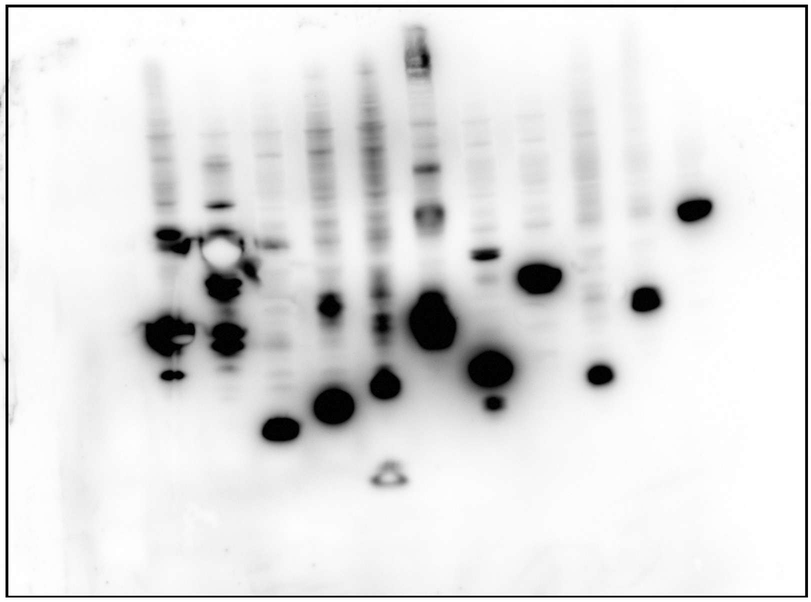

S3B

30sec

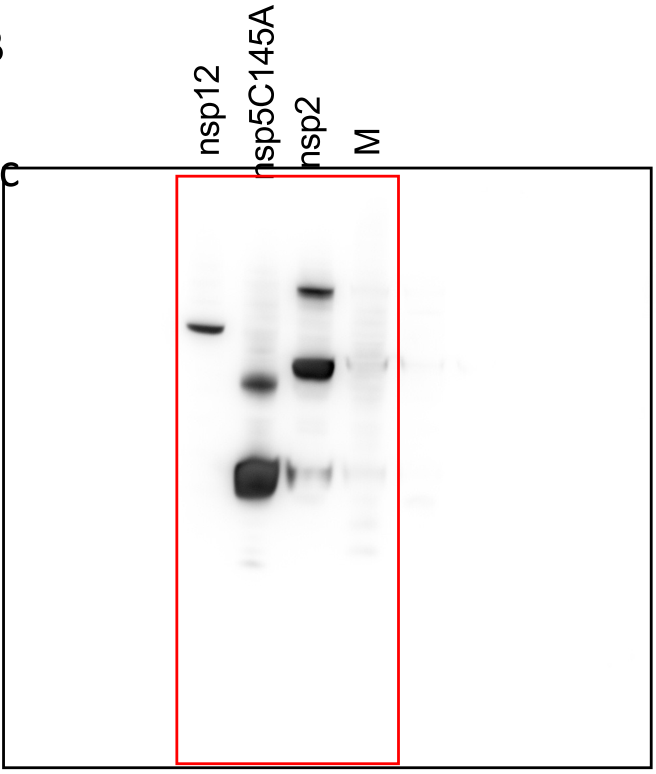

3min

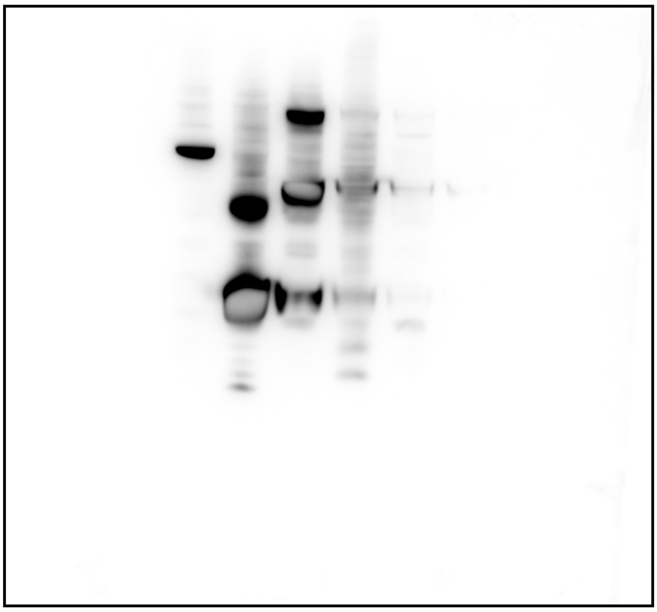

10min

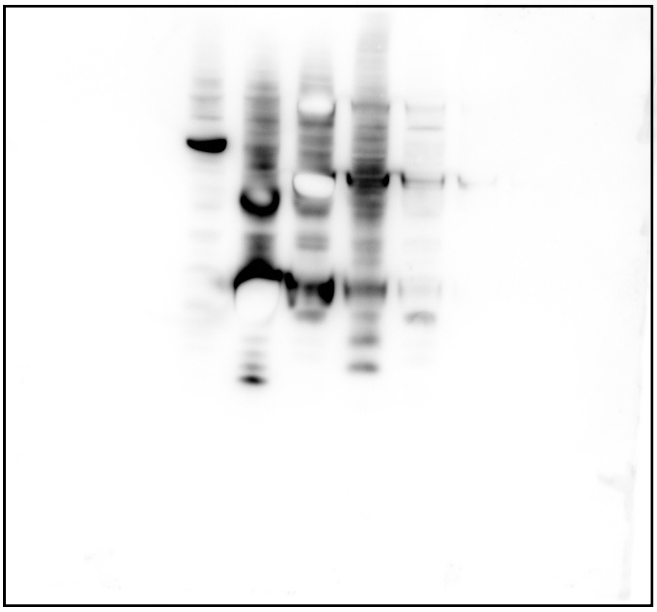

S3C

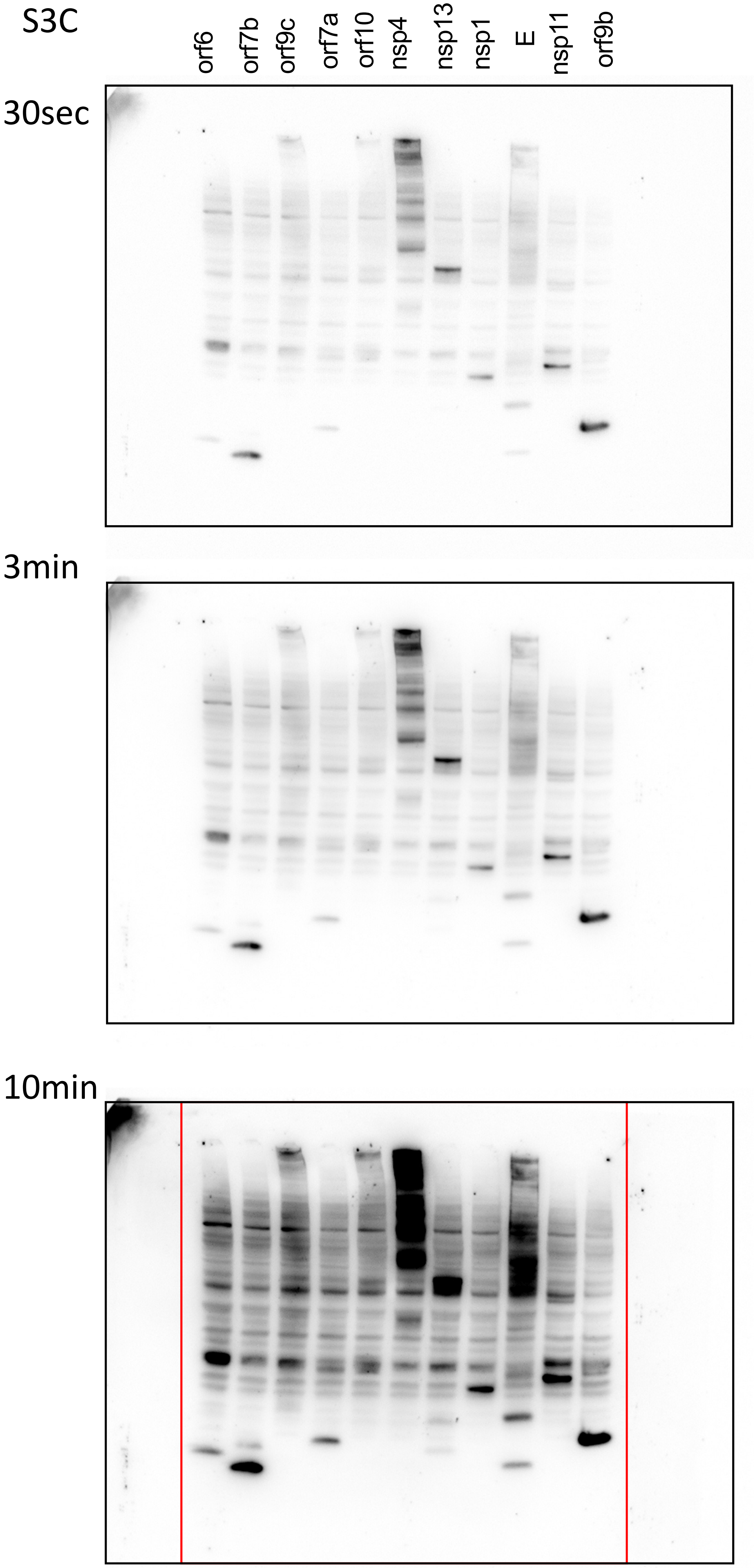
orf6
orf7b
orf9c
orf7a
orf10
nsp4
nsp13
nsp1
E
nsp11
orf9b
30sec
3min
10min

S3D

3min

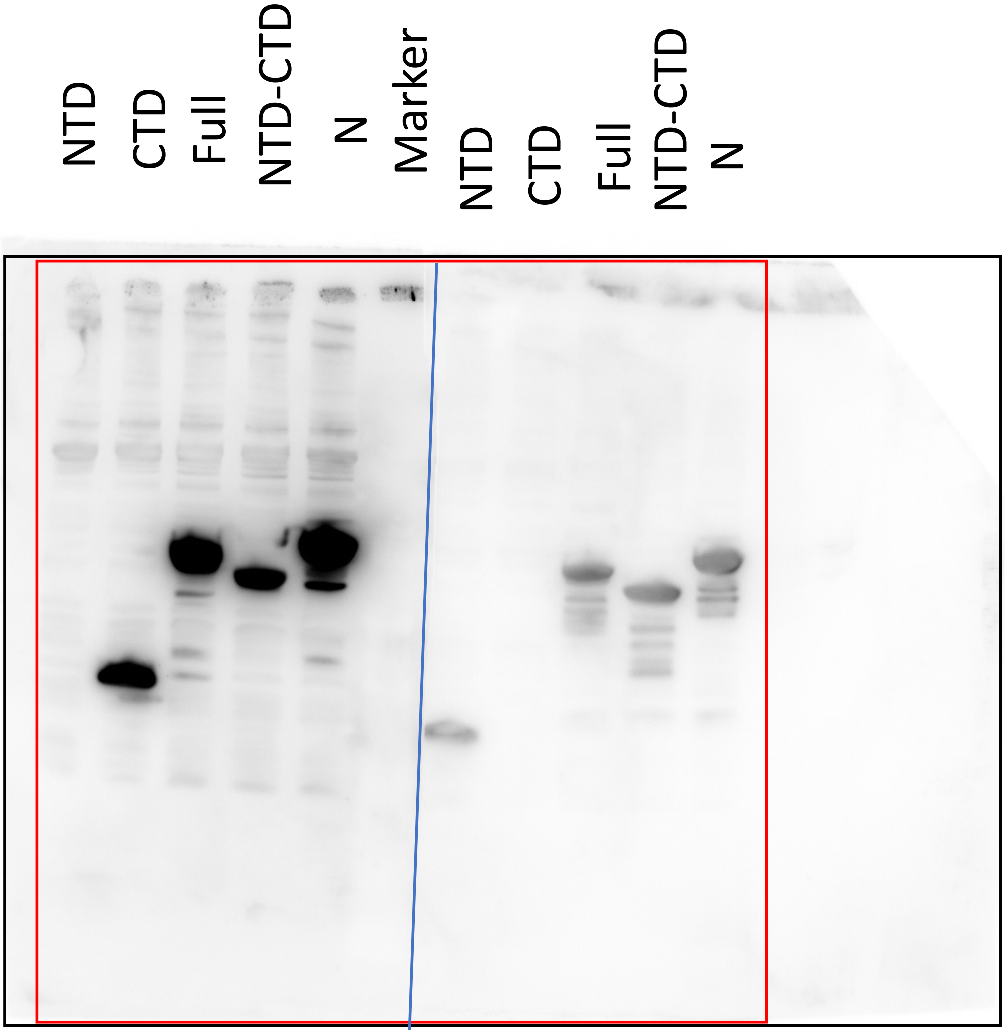

10min

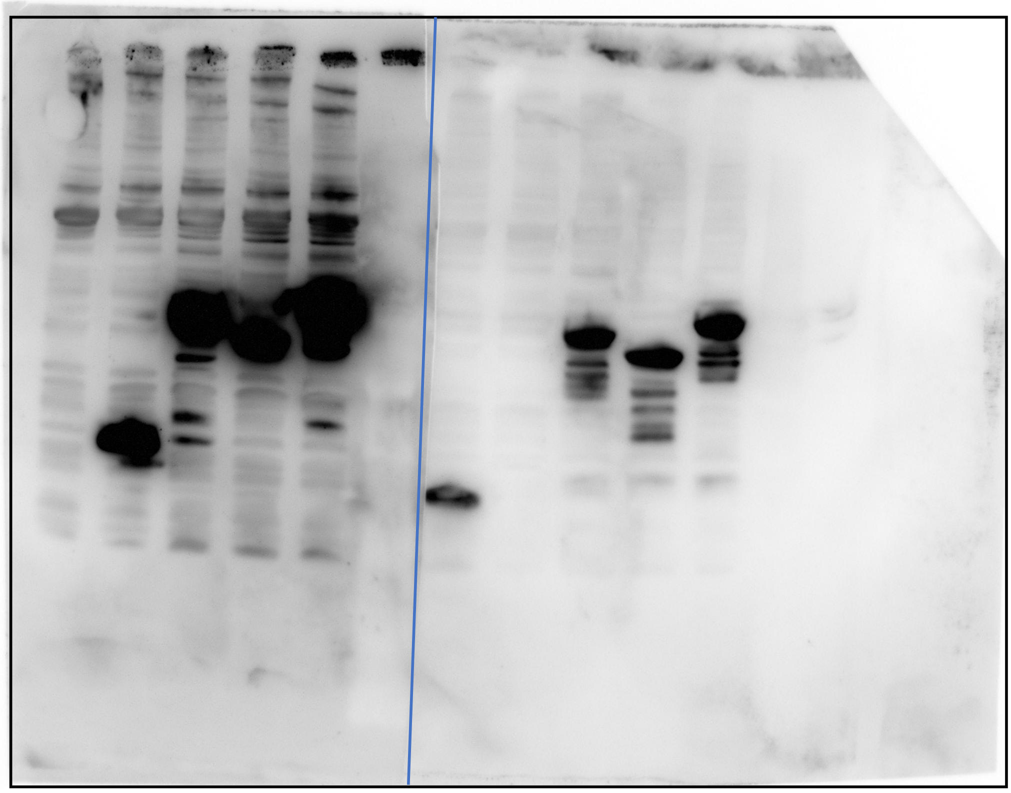

S4

A

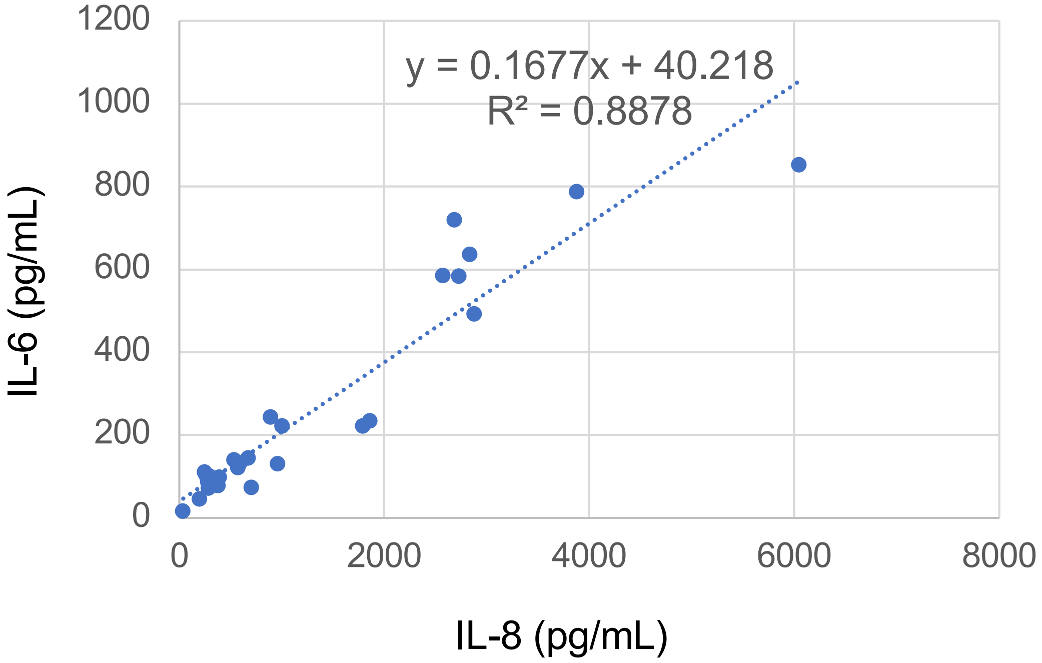

y = 0.1677x + 40.218
R² = 0.8878
1200
1000
800
600
400
200
0
0
2000
4000
6000
8000
IL-6 (pg/mL)
IL-8 (pg/mL)

B

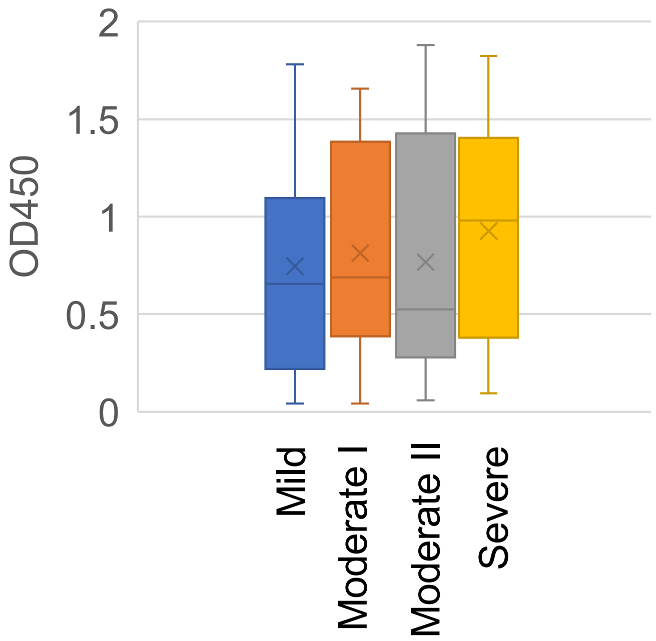

2
1.5
1
0.5
0
OD450
Mild
Moderate I
Moderate II
Severe

Figure S1

(A) TMPRSS2/VeroE6 cells and macrophages differentiated with GM-CSF were infected with 1000 50% tissue culture infectious dose (TCID50) or 100 TCID50 of SARS-CoV-2 (JPN-TY-Wk-521 strain) together with COVID-19 patient sera. Four hours later, cells were washed once. The viral RNA in the culture supernatants at days 2 and 6 was measured by RT-PCR. (B) iPS-derived myeloid cells K-ML2 (gray) and MDM (blue and red) were inoculated with the SARS-CoV-2 KNG-19-020 strain with SARS-CoV-2-infected or uninfected cell lysate. Four hours later, the MDM were washed once. Two days after infection, the IL-6 levels in the culture supernatants were measured by ELISA. (C) K-ML2 cells were stimulated with S protein for 2 days, and the IL-6 levels in the culture supernatants were measured by ELISA. The means and standard deviation of triplicate samples are shown.

Figure S2

TMPRSS2/VeroE6 cells were infected with the SARS-CoV-2 KNG19-020 strain, clinical isolate hCoV-19/Japan/OIPH14/2020 (A.1. lineage) or hCoV-19/Japan/OIPH21/2020 (B.1.1.214 lineage). On day 1, the cells were harvested and lysed by freeze-thawing. Macrophages differentiated with GM-CSF or M-CSF were infected with the SARS-CoV-2 KNG19-020 strain together with cell lysate and 0.5% serum from healthy donors. The negativity for SARS-CoV-2 infection of these healthy donors was confirmed by immunostaining of SARS-CoV-2-infected TMPRSS2/VeroE6 cells with these sera. After 4 h of incubation, the cells were washed once. The IL-6 levels in the culture supernatants at day 2 were measured by ELISA. The viral RNA in the remaining culture supernatants was measured by RT-PCR.

Figure S3

The original images of **w**estern blot assay presented in Figure 2B (A, B, C) and Figure 2E (D). The blotted membranes were developed for 30 sec, 3 min, and 10 min until the edges were visible. The black boxes denote the outline of the edges of the blots. The regions used for Figure 2B and 2E were denoted by red boxes. Blue vertical dividing lines of two membranes are shown in panel D.

Figure S4

(A) Elevated levels of IL-8 and IL-6 in the culture supernatant of K-ML2 cells treated with N protein (156 ng/mL) in the presence of 1% serum from 24 patients. The correlation between IL-8 and IL-6 levels is significant ($R=0.94$, $P<0.0001$).

(B) The levels of anti-N antibody in the patient sera were measured by an in-house ELISA. The center lines in the boxes and the boxes indicate the medians and 25/75 percentiles, respectively.
